# Benchmarking Mental Health Status Using Passive Sensor Data: Protocol for a Prospective Observational Study

**DOI:** 10.2196/53857

**Published:** 2024-03-27

**Authors:** Robyn E Kilshaw, Abigail Boggins, Olivia Everett, Emma Butner, Feea R Leifker, Brian R W Baucom

**Affiliations:** 1 Department of Psychology University of Utah Salt Lake City, UT United States

**Keywords:** audio data, computational psychiatry, data repository, digital phenotyping, machine learning, passive sensor data

## Abstract

**Background:**

Computational psychiatry has the potential to advance the diagnosis, mechanistic understanding, and treatment of mental health conditions. Promising results from clinical samples have led to calls to extend these methods to mental health risk assessment in the general public; however, data typically used with clinical samples are neither available nor scalable for research in the general population. Digital phenotyping addresses this by capitalizing on the multimodal and widely available data created by sensors embedded in personal digital devices (eg, smartphones) and is a promising approach to extending computational psychiatry methods to improve mental health risk assessment in the general population.

**Objective:**

Building on recommendations from existing computational psychiatry and digital phenotyping work, we aim to create the first computational psychiatry data set that is tailored to studying mental health risk in the general population; includes multimodal, sensor-based behavioral features; and is designed to be widely shared across academia, industry, and government using gold standard methods for privacy, confidentiality, and data integrity.

**Methods:**

We are using a stratified, random sampling design with 2 crossed factors (difficulties with emotion regulation and perceived life stress) to recruit a sample of 400 community-dwelling adults balanced across high- and low-risk for episodic mental health conditions. Participants first complete self-report questionnaires assessing current and lifetime psychiatric and medical diagnoses and treatment, and current psychosocial functioning. Participants then complete a 7-day in situ data collection phase that includes providing daily audio recordings, passive sensor data collected from smartphones, self-reports of daily mood and significant events, and a verbal description of the significant daily events during a nightly phone call. Participants complete the same baseline questionnaires 6 and 12 months after this phase. Self-report questionnaires will be scored using standard methods. Raw audio and passive sensor data will be processed to create a suite of daily summary features (eg, time spent at home).

**Results:**

Data collection began in June 2022 and is expected to conclude by July 2024. To date, 310 participants have consented to the study; 149 have completed the baseline questionnaire and 7-day intensive data collection phase; and 61 and 31 have completed the 6- and 12-month follow-up questionnaires, respectively. Once completed, the proposed data set will be made available to academic researchers, industry, and the government using a stepped approach to maximize data privacy.

**Conclusions:**

This data set is designed as a complementary approach to current computational psychiatry and digital phenotyping research, with the goal of advancing mental health risk assessment within the general population. This data set aims to support the field’s move away from siloed research laboratories collecting proprietary data and toward interdisciplinary collaborations that incorporate clinical, technical, and quantitative expertise at all stages of the research process.

**International Registered Report Identifier (IRRID):**

DERR1-10.2196/53857

## Introduction

### Background

Modern mental health care is the product of a tremendous volume of basic and applied research. Until recently, the majority of this research has relied on methods and practices (eg, randomized controlled trials [1]) that require large amounts of human labor to recruit participants (eg, through in-person recruitment at clinics or calls to members of research registries) and collect the necessary data (eg, through clinical interviews, performance-based tasks, or manual extraction from eHealth records). These methods and practices also tend to generate siloed data sets that are difficult to share beyond the original study team (see [2] for a review). Though existing research using these methods and practices has produced very valuable findings and greatly improved treatment options for individuals coping with mental health challenges, the pace of development is slow [3], and recent estimates suggest that only 40% to 50% of Americans who need mental health care receive treatment [4]. Novel approaches to mental health research are needed to address these challenges, and recent advances in computational analysis and digital technology have the potential to do just that (see [5-7] for reviews).

Computational psychiatry is an approach that includes both theory- and data-driven applications of mathematical modeling with the goal of advancing the diagnosis, mechanistic understanding, and treatment of mental health conditions [8-10]. For example, on the theory-driven side, reinforcement-learning models applied to functional magnetic resonance imaging data have helped predict posttreatment outcomes (ie, abstinence vs relapse) in patients with alcohol dependence [11], and Bayesian models of cognitive task performance have improved our understanding of how the ability to flexibly update previous beliefs differs between diagnoses (see [12] for a review). Similarly, data-driven studies using machine learning models with high-dimensional data (eg, electroencephalogram and magnetic resonance imaging data) have been able to accurately distinguish patients with schizophrenia from controls [13,14] and have also identified multivariate “biomarkers” that have helped improve pharmacological treatment-response prediction in patients with depression [15-17].

Enthusiasm about this body of work has led to numerous calls to extend these methods to mental health risk assessment in the general public [18-20]. However, one of the largest barriers to doing so is that many of the data sources used in computational psychiatry research with clinical samples (eg, brain imagery data and standardized task performance data) are not available for community-dwelling individuals in the general population. As a result, researchers are increasingly leveraging the near ubiquity of personal digital devices (PDDs) [21] (eg, smartphones and smartwatches) as a more scalable and accessible means of collecting high-dimensional behavioral, contextual, and even physiological data streams from individuals [22,23]. Research of this type represents a subset of the broader computational psychiatry field commonly referred to as digital phenotyping.

Digital phenotyping refers to using quantitative methods with PDD data—particularly passive sensor data (eg, GPS, accelerometry, call and text logs, and app usage)—to identify behavioral phenotypes or “digital biomarkers” relevant to mental health [24-26]. Thus far, digital phenotyping research has primarily focused on using computational methods such as machine learning models for detecting, monitoring, and predicting changes in symptom severity as well as predicting or improving treatment response in clinical samples (for reviews, see [27-29]). For example, machine learning algorithms applied to passive sensor data have been able to successfully identify depressive and manic episodes in individuals with bipolar disorder [30,31] and predict psychotic relapses in patients with schizophrenia [32]. Furthermore, digital biomarkers derived from PDD data have demonstrated reasonable accuracy for predicting treatment response to transcranial magnetic stimulation in patients with depression [33]. Finally, a growing body of research demonstrates that passive sensor data from PDDs can also be used to identify common risk factors for mental health conditions (eg, stress, depressed mood, and anxiety) in clinical [34] and student samples [35].

This nascent body of research provides an empirical and methodological foundation for extending digital phenotyping to identify markers of mental health risk in the general population. While, to the best of our knowledge, no studies have done this using a prospective study design with community-dwelling adults, existing computational psychiatry and digital phenotyping research, along with guidance from leading advocates in these fields, provides a strong set of recommendations for generating a data set that is well-suited to this objective. These recommendations include: (1) using a transdiagnostic and dynamic understanding of mental health to guide study design and data collection [20,36]; (2) incorporating the data requirements of cutting-edge computational methods into data collection methods [37,38]; and (3) using careful consent procedures and data curation methods to ensure that data can be safely and ethically shared with researchers across academia, industry, and government so as to harness the expertise of diverse professionals working toward improving mental health [39].

### Objectives

On the National Institute of Mental Health Data Archive [40], there currently exists a small number of shareable computational psychiatry data sets that include longitudinal PDD data from community-dwelling participants [41,42], while other such data sets are in the process of being created [43,44]. Nevertheless, all of these data sets comprise participants who either meet specific diagnostic criteria (eg, trauma exposure [42], diagnosis with a serious mental illness [43], and binge drinking [44]) or developmental characteristics (eg, school-attending adolescents) and are best suited for identifying digital biomarkers of mental health risk in clearly defined subsamples of the population. Therefore, as a complement to this existing work, we aim to create the first computational psychiatry data set that is tailored to studying mental health risk in the adult general population, includes PDD sensor-based behavioral features, and is designed to be widely shared across academia, industry, and government using gold standard guidelines for privacy, confidentiality, and data integrity*.*

To maximize the relevance and use of this data set for researchers from a wide array of disciplines, we will use the guidelines listed above to ensure our proposed data set is optimized for both mental health and computational considerations. On the mental health side, our proposed data set will be informed by state-of-the-art etiological and phenomenological models of mental health and mental health disorders. On the computational side, our proposed methods are designed to generate a high-dimensional, multimodal, and multirate feature set with maximum variability across levels of measurement and analysis, a balanced classification design, and minimal missing data [22,38]. These considerations will therefore support the primary objective of this project, which is to create a data set that, through its design and accessibility, has the potential to advance mental health risk assessment in the general population using digital phenotyping methods. Although we do not have specific hypotheses or analytic plans guiding the creation of this data set, we believe that in combination with computational methods, the data we collect could be used to investigate research questions such as the following: can PDD sensor-based features predict the likelihood of a future mental health event (eg, receiving a psychiatric diagnosis or treatment) at rates significantly above chance? and does incorporating information about preexisting vulnerability factors (eg, difficulties with emotion regulation and past mental health conditions) improve the accuracy of these predictions?

## Methods

### Participants

We are recruiting 400 individuals at varying levels of risk for a lifetime incidence of experiencing an episodic mental health disorder (eg, depression, anxiety, and adjustment disorder). To achieve this goal, we are using a stratified, random sampling design with 2 crossed factors: (1) difficulties with emotion regulation [45], a transdiagnostic risk factor for a wide range of mental health conditions [46], and (2) perceived overall life stress during the past 30 days [47]. These 2 factors are assessed during screening, and eligible individuals will be included in the sample such that 40% to 60% of participants report higher than average difficulties regulating strong emotions and 40% to 60% report currently experiencing significant life stress. This sampling design and the choice of these 2 factors were guided by the diathesis-stress model of psychopathology, which suggests that many episodic (ie, non-neurodevelopmental [48]) psychological disorders are the result of an interaction between preexisting vulnerabilities—in this case, difficulties with emotion regulation—and stress due to life experiences [49]. Participants must also meet the following eligibility criteria: be 18 years of age or older, currently living in Utah, have a smartphone with an active cellular data plan and an Apple or Android operating system, be able to speak and read English fluently, and receive their health care through either the University of Utah Health (UHealth) or Intermountain Health Care (IMHC) systems.

Individuals who report suicidal ideation in the past 3 months or any history of a suicide attempt, active mania, or psychosis (ie, severe mental health problems potentially requiring hospitalization during their participation) during screening are ineligible to participate. Initially, individuals who reported current symptoms of a substance use disorder (eg, daily binge drinking) and were not engaged in substance use treatment were also ineligible for participation; however, we received additional funding after beginning the study that allowed us to increase the study staffing so that we could remove this exclusion criterion. This change to the exclusion criteria occurred approximately 6 months into recruitment, after 180 participants—almost half of our target sample size of 400—had provided consent. Finally, individuals with a history of conviction for a violent crime; a history of child abuse or neglect perpetration substantiated by child protective services; and, for individuals in committed relationships, recent physical intimate partner violence are also excluded for potential ethical reasons. Specifically, licensed mental health providers and physicians are legally required to report instances of each of these final criteria to authorities in most states in the United States, and this duty is not waived by a certificate of confidentiality. The presence of these events would therefore render the data set unshareable for most purposes.

### Procedures

Participants are recruited using web-based advertising through the University of Utah websites, social media websites, and listserves, as well as paper fliers posted throughout the Salt Lake City area. Interested participants complete a web-based screening survey to determine eligibility. Eligible participants are then forwarded to an electronic consent form, and consenting participants complete another web-based battery of questionnaires to assess current and lifetime psychiatric and medical diagnosis and treatment, current psychological symptoms and social functioning, and demographic variables. Participants who complete this baseline battery are compensated US $25 and are scheduled for a videoconference call during which study staff instructs them on how to use the study equipment (ie, audio recorder) that is mailed to them.

Following this call, participants begin a continuous 7-day period of intensive data collection that includes wearing the audio recorder during waking hours, providing raw sensor data collected through a smartphone app (Beiwe [50]), completing a brief survey to assess mood and important events at the end of each day, and responding to a brief phone call from study staff every evening. During this phone call, study staff assess compliance with study procedures, ask participants to describe the most positive and negative events from that day, and inquire whether there is any period of the recording from that day that they wish to delete. Providing participants the opportunity to review the contents of their recordings is necessary to meet ethical standards for long-term, ambulatory data collection [51]. After returning the audio recorder, participants are compensated US $10 and US $4.28 for every day that they provided raw sensor (PDD) data and audio recording, respectively.

**Figure 1:**
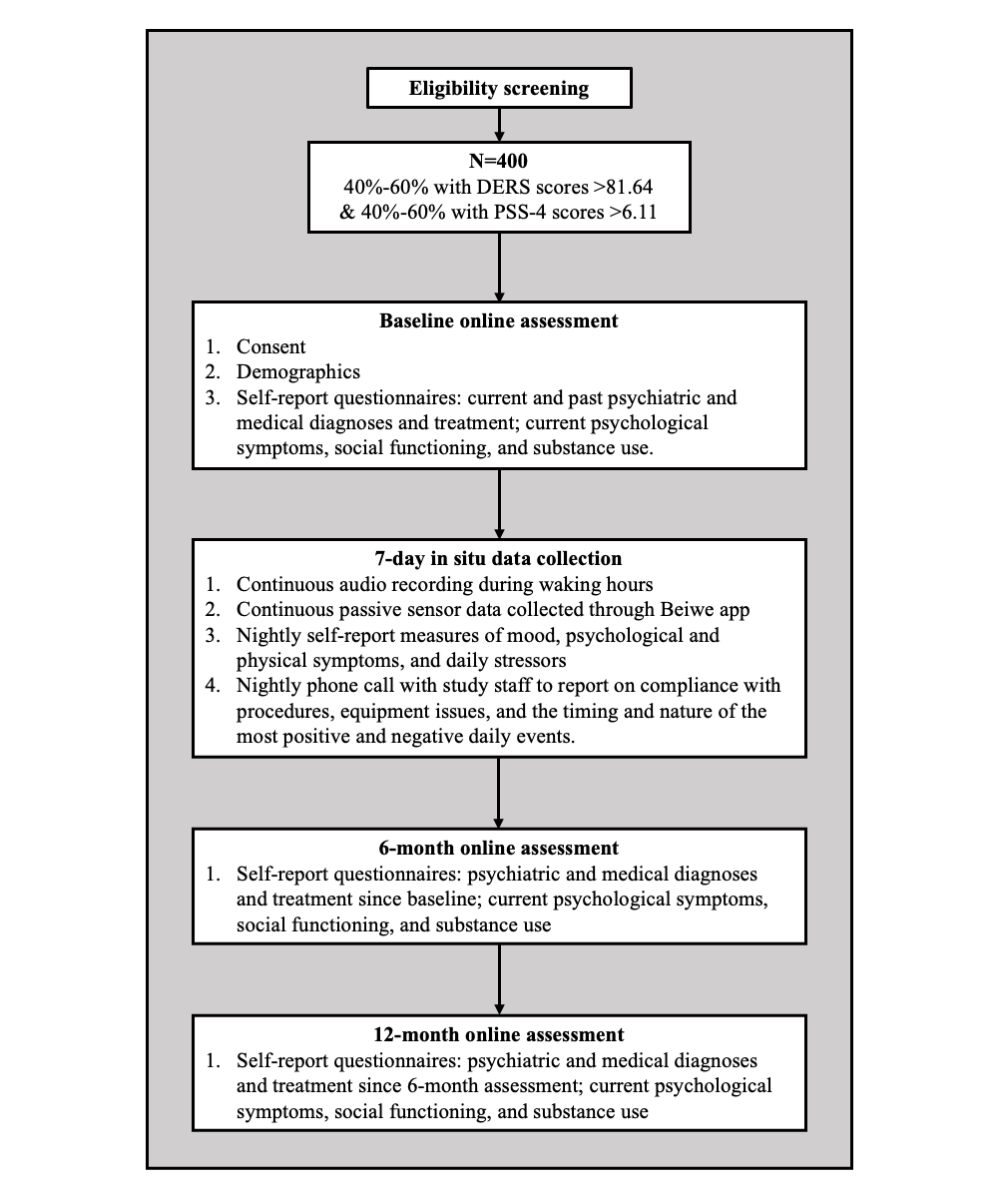
Procedures flowchart. DERS: Difficulties with Emotion Regulation Scale; PSS-4: Perceived Stress Scale-4.

Participants also complete web-based questionnaires 6 and 12 months after the 7-day intensive data collection phase to reassess current psychological symptoms and social functioning, as well as psychiatric and medical diagnosis and treatment during the intervening time; they are compensated US $20 for the completion of each of these questionnaires. See Figure 1 for the complete procedure flow diagram. All of these procedures are described in more depth below.

### Data Privacy and Confidentiality

To maximize data privacy and security, data from all sources are encoded and can only be matched using a key maintained in a password-protected file only accessible to approved study personnel. Study data collected from the Beiwe app are encrypted in transit and at rest, and no identifiable data are stored on participants’ devices. For complete details on all the security features of the Beiwe research platform, see [52]. Audio files are saved to a microSD card and returned with the recorders through registered mail. All digital data, both in raw and processed formats, are stored in a Health Insurance Portability and Accountability Act (HIPAA)–compliant protected environment maintained by the University of Utah Center for High-Performance Computing.

### Data Sources

#### Self-Report Questionnaires

Participants are given the option to skip any self-report questionnaire item. See [53] for full versions of all publicly available standardized measures as well as any nonstandardized questionnaires included in this study (ie, demographics questionnaire, clinical history questionnaire, and daily events questionnaire).

#### Difficulties With Emotion Regulation Scale

The Difficulties with Emotion Regulation Scale (DERS) [45] is a well validated and widely used measure of subjective emotion regulation ability [54]. A total of 36 items are responded to on a 5-point Likert scale (from 1=“almost never [0%-10%]” to 5=“almost always [91%-100%]”; range of scores 36-180), such that higher total scores indicate greater difficulty regulating in the context of strong emotions. Overall, 6 subscale scores can also be generated, capturing individuals’ lack of emotional awareness, lack of emotional clarity, nonacceptance of emotions, limited access to emotional regulation strategies, and difficulties engaging in goal-directed behavior or inhibiting impulsive responses in the context of strong emotions. The DERS is administered as part of the screening survey and is only presented to individuals who have already met all other inclusion and exclusion criteria. The mean total DERS score from a community sample [55] is used to classify participants as having high (ie, total score >81.64) versus low (ie, total score ≤81.64) difficulties with emotion regulation for enrollment purposes.

#### Perceived Stress Scale-4

The Perceived Stress Scale-4 (PSS-4) [47] is a 4-item short form of the widely used Perceived Stress Scale, which measures individuals’ subjective level of life stress during the previous month. The PSS-4 displays acceptable psychometric properties in nonclinical populations [47,56] and is used to assess how unpredictable and overwhelming individuals currently find their lives. Items are responded to on a 5-point scale anchored by 0 (never) and 4 (very often), and higher total scores indicate greater perceived stress (range of scores 0-16). The PSS-4 is only presented to eligible participants as part of the screening survey. The mean total score from a validation sample of community participants [56] is used to identify participants experiencing high (ie, total score >6.11) versus low (ie, total score ≤6.11) life stress for enrollment purposes.

#### Demographics Questionnaire

As part of the baseline battery, participants are asked to report on a number of demographic factors, including age, biological sex, gender, sexuality, relationship status, race, ethnicity, spoken languages, religion, education history, and current employment and income.

#### Clinical History Questionnaire

Participants are asked to provide information about their current and past physical and mental health, including smoking status; history of a major medical event (eg, cancer, diabetes, or stroke); current and past psychiatric diagnoses; current and past use of psychiatric medication; or other mental health treatment, including when and what service or medication was used. This questionnaire was created for this study and is included in the baseline battery as well as the 6- and 12-month follow-up questionnaires as a way of assessing any significant physical or mental health changes during the study period.

#### Depression, Anxiety, and Stress Scale-21

The Depression, Anxiety, and Stress Scale-21 (DASS-21) [57] is a 21-item questionnaire designed to measure depression, anxiety, and tension or stress during the past week. It is a short form of the widely used and well-validated original DASS-42 [58]. Individuals respond to items on a 4-point Likert scale from 0 (did not apply to me at all) to 3 (applied to me very much or most of the time), and 3 subscale scores are produced corresponding with the 3 negative emotional states. The DASS-21 is administered at baseline as well as at the 6- and 12-month time points.

#### Tobacco, Alcohol, Prescription Medications, and Other Substance Tool

The Tobacco, Alcohol, Prescription Medications, and Other Substance (TAPS) [59] tool is a 2-part measure of substance use. In the first part, individuals respond to 4 items assessing how frequently (ranging from “never” to “daily or almost daily”) they have used tobacco, alcohol, illicit drugs, or prescription drugs for nonmedical reasons in the past year. Any individual who screens positive in part 1 (ie, responds with anything other than “never”) then completes part 2, which consists of a brief assessment of use-related behaviors during the past 3 months. Scores from part 2 can be used to generate 3 levels of risk for each substance endorsed (ie, no use in the past 3 months, problem use, and higher risk). The TAPS tool has demonstrated adequate psychometric properties as a screening measure for high-risk substance use behaviors in adult primary care patients [59]. We are including a measure of substance use in our data set because this is a well-established, transdiagnostic behavioral marker of risk, and in combination with our affective marker of risk (ie, difficulty regulating emotions), it will improve the precision of risk estimation [60]. This measure is administered at baseline as well as at the 6- and 12-month time points.

#### Life Functioning Questionnaire

The Life Functioning Questionnaire (LFQ) [61] is a 2-part questionnaire designed to assess individuals’ subjective difficulty functioning in 4 domains of life (leisure time with friends, leisure time with family, duties at work or school, and duties at home) during the past month. In part 1 of the LFQ, individuals indicate the degree of problems (from 1 [no problems] to 4 [severe problems]) they have experienced within each domain in terms of the amount of time spent on related activities, amount of conflict experienced, level of enjoyment, and self-assessed performance (for work and home duties only). For duties in the work or school domain, individuals are also asked to indicate the number of days they were absent as well as the factors that contributed to their absence (eg, mental or physical health symptoms and interpersonal difficulties). Part 2 asks additional questions about individuals’ work situation during the previous month, previous full-time work, and reasons for leaving, as well as their living and financial status in the past 6 months. The LFQ was originally designed to assess functional capacity in psychiatric patients and has demonstrated adequate psychometric properties with adult inpatients seeking treatment for mood disorders [61]. In this study, the LFQ is administered at baseline and at the 6- and 12-month time points.

#### Brief Symptom Inventory-18

The Brief Symptom Inventory-18 (BSI-18) [62] is an 18-item measure that assesses the level of distress a person has experienced in the past day due to various psychological (eg, feelings of worthlessness) and physical (eg, pains in the heart or chest) symptoms. Questions are responded to on a 5-point scale from 0 (not at all) to 4 (extremely) and can be summed to produce 3 subscale scores (depression, anxiety, and somatization) as well as a global severity index that measures overall psychological distress. The BSI-18 has been validated and normed with community samples and is acceptable to use repeatedly as a measure of symptom change [63]. The BSI-18 is administered nightly during the 7-day intensive data collection phase of this study.

#### Daily Positive and Negative Mood Questionnaire

Aspects of participants’ daily mood are assessed using a version of the Positive and Negative Affect Schedule [64] described by Smyth et al [65]. Participants rate their current level of 4 positive and 5 negative mood adjectives on a 7-point scale anchored by 0 (not at all) and 6 (extremely). Items are summed to produce positive and negative mood subscales that demonstrated acceptable psychometric properties in a previous sample of community participants [65]. This measure is administered nightly during the 7-day intensive data collection phase of this study.

#### Daily Events Questionnaire

The Daily Events Questionnaire asks individuals to select from a list of common daily stressors (eg, a lot of work at school or work or a financial problem) and people (eg, a friend or spouse) to assess if any troublesome events happened to them since they woke up that morning, as well as if they experienced any tension or arguments with anyone. Individuals can select as many options as apply and have the option to describe any “other” event or relationship not listed. For each selected event, individuals are then asked to indicate approximately when this event occurred and how distressed they felt during the event, from 1 (not at all) to 10 (very much). Similarly, for each person that a participant indicates they had an argument with, they are asked to provide approximately when the argument began and ended, as well as how distressed they felt and how satisfied they felt with the outcome of the event (using the same 10-point response scale). This measure has been used previously to successfully identify the approximate timing of various distressing events throughout the day [66]. In this study, it is administered nightly during the 7-day intensive data collection phase of this study.

#### Daily Call With Study Staff

Study staff calls participants each evening near the end of the day (ie, between 6 PM and 8 PM) to inquire about compliance with study procedures, problems with study equipment, and the nature and timing of participants’ most positive and negative events of the day. A transcript of the semistructured interview questions used during these phone calls is available on our Open Science Foundation site [53].

#### Audio Data

In situ audio is continuously recorded while participants are awake at 24-bit/48 kHz using omnidirectional Lavalier microphones connected to miniature field recorders. Recordings are segmented into smaller, 15-minute-long files to optimize data transfer and increase data processing efficiency.

We have carefully considered legal and ethical issues in proposing these in situ recordings. Audio recordings are governed by wiretapping laws, which vary from state to state. Utah is a single-party consent state, meaning that as long as a study participant consents to be recorded, other individuals captured on those recordings do not need to additionally consent. However, the ethical principles of beneficence, nonmaleficence, autonomy, and justice require that other individuals who may be recorded need to be aware of that possibility and given the opportunity to not be recorded. For these reasons, participants are instructed to wear a badge that states that they are participating in a study that records audio during daily life and will be allowed to pause the recording whenever an individual they come into contact with requests that they do so, or the participant wishes or is required to do so by policy or law [51]. We have used these methods in previous work, and they typically generate 10 to 14 hours of audio per day.

#### Passive Sensor Data

Raw smartphone sensor data are collected using Beiwe, a cross-platform digital phenotyping app created by Onnela and colleagues [25,50]. The Beiwe platform collects raw data generated by smartphone sensors, including, but not limited to, GPS and accelerometry, Wi-Fi connectivity, Bluetooth device scans, phone and screen status, and phone call and SMS text message event logs linked to onboard device contacts. The specific data collected for a participant is determined by the sensors on their smartphone and the policies of the smartphone manufacturer [52].

#### Medical Records

The state of Utah maintains the Utah Population Database (UPDB) and All Payer Claims Database, which are standardized (ie, use the Systemized Nomenclature of Medicine–Clinical Terms), digital archives of medical encounters in the UHealth and IMHC systems, and insurance claims filed in the state, respectively. These databases are generated for research purposes and will be used to verify and augment participant reports. These databases represent a highly unique and valuable opportunity because it is well documented that retrospective reports of psychiatric history incorrectly fail to identify ~25% to 40% of true positives relative to medical records [67]. Relevant records from these databases will be linked to other participant data. UPDB policy dictates that these records will not be shareable, and individuals or entities wishing to access them will have to seek permission directly from the database administrators.

### Planned Data Processing

Raw item responses as well as relevant total and subscale scores from the self-report questionnaires will be created using standard scoring protocols and included for all self-report measures in the final data set. In addition to raw DERS scores, age- and gender-adjusted *t*-scores computed using the methods in [55] will be included in the final data set. Annotations of participants’ most positive and negative daily events will also be available in the data set. To create these, study staff will use the information provided by participants during the daily phone calls to annotate details about their most positive and negative event for each day, including the beginning and end time of the event, and the type (eg, interaction with another person or financial problem) and nature of the event (eg, positive vs negative). Study staff will additionally annotate speaker IDs (eg, participant and female 1), emotional expressions, and communication behaviors (eg, arguing or cooperating) using information from the corresponding recorded audio.

Audio recordings will be processed to generate gold-standard acoustic feature sets used in behavioral signal processing [68] and affective computing [69] research using the openSMILE toolkit [70]. These methods produce 88 acoustic variables that represent frequency-, energy-, and spectral-related aspects of speech and ambient noise. Acoustic variables will be generated over the smallest window of time possible for each variable and downsampled to produce a summary score for each acoustic variable for each 1 second of the recording.

Raw PDD sensor data will be processed using Forest [71], a freely available library for analyzing Beiwe data developed by the creators of Beiwe. Similar to the acoustic variables described above, Forest produces summary variables for each sensor type that quantify a wide range of behavioral and contextual information. For example, outputs of GPS data include time spent at home, total distance traveled, physical circadian rhythm, and the type (eg, shop, restaurant, and place of worship) and duration of locations visited. The location type is generated using information from the open-source platform OpenStreetMap [72] and is particularly valuable for the current data set as it will allow for the creation of a library of geographically referenced place tags that index risky (eg, amenity=bar; amenity=tobacco retailer) and protective (eg, amenity=library; amenity=gym) locations and will increase the potential information value of the database by providing additional context for the passive sensor data streams collected. Another example of the summary features available from Forest includes outputs of call and text logs, the total number of calls received, the total number of unique callers, and the total duration of calls received. Additional details about all summary variables are available on the Forest GitHub page [71]. Summary variables will be generated for each day and included in our final data set.

### Ethical Considerations

All study procedures were approved by the University of Utah Institutional Review Board (00149365). Eligible participants are given the opportunity to review all study procedures, including planned data-sharing processes, and are provided with contact information for the study’s principal investigator to answer any additional questions before signing the consent form on the internet. Several measures are in place to protect the privacy and confidentiality of participants’ data (see *Data Privacy and Confidentiality* and *Results* sections for additional details). These include using a secure and password-protected database for storing all study data, only sharing deidentified and nonsensitive data publicly, and requiring a more stringent data use agreement and relevant ethics approval to be provided by individuals requesting access to identifiable data sources (eg, raw audio data). All participants are offered US $165 as compensation for completing all study procedures.

## Results

Data collection for this project began in June 2022 and is expected to conclude by July 2024. To date, 310 participants have consented to the study; 149 have completed the baseline questionnaire and 7-day intensive data collection phase; and 61 (ie, 85% of eligible participants) and 31 (ie, 91% of eligible participants) have completed the 6- and 12-month follow-up questionnaires, respectively.

Once completed, the proposed data set will be made available consistent with the findable, accessible, interoperable, and reusable guidelines for data management and stewardship [73]. We will publish a description of the data set in a general science outlet with a broad readership to increase awareness of it among a broad audience. We will also index the data set on recommended data banks, such as the Open Science Foundation and Science Data Bank [74] to increase its findability.

We will use a stepped approach to making the data set accessible to academic researchers, industry, and government. Deidentified, nonsensitive data (eg, raw item and scale scores from self-report measures, summary features from acoustic and passive sensor data, and annotations of daily events) will be publicly available for download directly from data repositories after completion of a brief data use agreement but without contact with the study team. Identifiable and other sensitive data (eg, raw audio recordings and raw passive sensor data) will only be made available for download from a University of Utah server after researchers have provided evidence of the necessary approvals from their institutional review board or comparable entity, completed a more stringent data use agreement, and have communicated with a member of the study team. Data will be made available to academic and government researchers at no cost and licensed to commercial entities.

## Discussion

### Contributions

Computational psychiatry and digital phenotyping have the potential to make significant contributions to mental health research and treatment development. Digital phenotyping studies have already demonstrated that by applying machine learning techniques to PDD data, it may be possible to predict upcoming mental health events (eg, manic episodes and hospitalization) in clinical samples [32] and detect mental health risk factors (eg, increasing stress and depressed mood) in students [35]. These findings are paving the way for a future where digital markers of increased risk could be used to trigger just-in-time interventions that connect individuals with their mental health care team when they need support most and before their symptoms worsen [75]. Similarly, ongoing computational psychiatry work aims to improve mental health treatment by using machine learning to identify the precise cognitive and neurobiological mechanisms underlying psychiatric disorders and their symptoms [76]. These findings may then be used to gain a better understanding of which treatments work best for which patients at what time.

Whereas much of this existing work focuses on predicting and treating mental health symptoms in individuals who meet certain diagnostic criteria or have received treatment for a psychiatric diagnosis, we are proposing a data set that is designed to advance the field’s ability to identify community-dwelling members of the adult general population at risk for a future mental health event. This is a complementary approach to current computational psychiatry and digital phenotyping research and has the potential to improve mental health risk assessment within the general population. The proposed data set possesses several strengths that make it well suited for this goal. First, in response to numerous calls for more theory-driven digital phenotyping studies [20], both the sample we are recruiting and the data we are collecting are guided by a well-established, transdiagnostic model of psychopathology development—the diathesis-stress model [49]. Second, instead of focusing on stable individual differences or 1-time measures of risk, the proposed data set will include multimodal data collected across multiple time scales and levels of analysis. By including digital traces of behavior that can be linked to both time and context, the proposed data set will therefore allow for the identification of features that better capture the dynamic nature of mental health risk [36,37]. Third, by using the Beiwe platform to collect raw PDD data, future analyses will not be limited by inconsistencies in the algorithms used to derive summary statistics or features from different devices [22]. Similarly, including raw PDD data in this data set means that not only is it designed to support current computational techniques, but it will continue to be relevant for future quantitative developments. Finally, the shareable nature of this data set will encourage interdisciplinary collaboration and ideally maximize the rate, rigor, and accuracy of the predictive machine learning models that are developed from it.

### Limitations

Although the proposed data set possesses many strengths, there are also some limitations to acknowledge. First, the types of digital data included in this data set are limited to what is available through the Beiwe platform [52]. Although it is possible that other passive data streams (eg, app usage log) may carry digital traces of participants’ behaviors that are informative for mental health prediction, our decision to use only what is available from Beiwe was guided by the desire to prioritize participant privacy and the shareability of the data set, which are 2 features supported by the design and maintenance of Beiwe. Second, some of the data sources captured by Beiwe are not consistent across Apple and Android devices due to variability in the sensors installed on different devices and the policies of different smartphone manufacturers (eg, call and SMS text message logs are only available on Android devices [52]). This may limit some of the analyses that can be performed and features that can be developed if certain data sources are only available from a small subset of the total sample. Third, our decision to exclude participants with suicidal thoughts and behaviors or other persistent and severe mental health conditions (ie, active mania or psychosis) means that the predictive models arising from this data set will not be optimized for identifying an increase in risk for the mental health events associated with these conditions (eg, inpatient hospitalization). However, such events are less likely in the general population and are not the primary outcome of interest for the proposed data set.

### Conclusions

Computational psychiatry and digital phenotyping have been lauded as pillars of the next great revolution in mental health care [7,8,39]. The past 2 decades have seen a dramatic increase in the number of mental health studies using these methodologies with PDD data. Furthermore, rapid developments in digital technology and quantitative analysis suggest that the potential benefits of PDD data and computational techniques for mental health research and treatment development are only going to continue to expand. In order to achieve these benefits, it will be important for the field to move away from siloed research laboratories collecting proprietary data and toward interdisciplinary collaborations that use clinical, technical, and quantitative expertise to produce widely applicable and shareable data sets.

## Abbreviations

BSI-18: Brief Symptom Inventory-18
DASS-21: Depression, Anxiety, and Stress Scale-21
DERS: Difficulties with Emotion Regulation Scale
HIPAA: Health Insurance Portability and Accountability Act
IMHC: Intermountain Health Care
LFQ: Life Functioning Questionnaire
PDD: personal digital device
PSS-4: Perceived Stress Scale-4
TAPS: Tobacco, Alcohol, Prescription medications, and Other Substance
UHealth: University of Utah Health
UPDB: Utah Population Database

## Data Availability

Upon completion of this study, the data sets generated and analyzed during this study will be available in a widely accessible data repository.
